# Mutual adaptations between hosts and parasites determine stress levels in eels

**DOI:** 10.1016/j.ijppaw.2021.02.001

**Published:** 2021-02-20

**Authors:** K.I. Honka, B. Sures

**Affiliations:** a Aquatic Ecology, University of Duisburg-Essen, D-45141, Universitätsstr. 5, Germany; b Center for Water and Environmental Research, University of Duisburg-Essen, D-45141, Universitätsstr. 5, Germany

**Keywords:** Cortisol, Stress response, *Anguilla Anguilla*, *Anguilla japonica*, *Anguillicola crassus*, *Pomphorhynchus*, *sp.*

## Abstract

Invasive parasites may severely affect their new hosts. Two invasive parasites occurring in the European eel (*Anguilla anguilla*) are the Asian swim bladder nematode *Anguillicola crassus* and the Ponto-caspian acanthocephalan *Pomphorhynchus* sp., which were introduced to the river Rhine in the early 1980/90s. The Japanese eel (*Anguilla japonica*), as the native host of *A. crassus*, developed mutual adaptations to the swim bladder parasite, which are lacking in the European eel. Therefore, after its spread to Europe, infestations of European eels with *A. crassus* were found to be more severe and caused massive swim bladder wall damages mainly due to the feeding activity of the adult nematodes. A suppression of the immune system also appears to be likely, which allows secondary infections e.g. by bacteria or other parasites in European eels. Acanthocephalans of the genus *Pomphorhynchus* have not been described so far in Japanese eels, in contrast to European eels, which regularly show infestations with *Pomphorhynchus* sp. By using these differentially adapted host-parasite associations for experimental studies, host stress responses were examined in the present study in relation to the degree of mutual adaptations between eel hosts and parasites.

Under laboratory conditions, Japanese and European eels were each inoculated with *A. crassus* and *Pomphorhynchus* sp., respectively, to investigate their stress responses against differently adapted parasites. The stress response was determined by analyzing plasma levels of cortisol, which is the main corticosteroid hormone during stress response of fish. The results show a strong cortisol release in European eels after infestation with *A. crassus* whereas Japanese eels only react against *Pomphorhynchus* sp. infestations. These results are consistent with the initial hypothesis that a low degree of host-parasite adaptations lead to stronger host stress responses against the parasite.

## Highlights

• We measured cortisol as the main corticosteroid hormone in fish.
• Japanese and European eels were inoculated with *A. crassus* and *Pomphorhynchus* sp.
• Lower mutual adaptations lead to a higher stress response in host-parasite-systems.

## Graphical abstract

**Figure:**
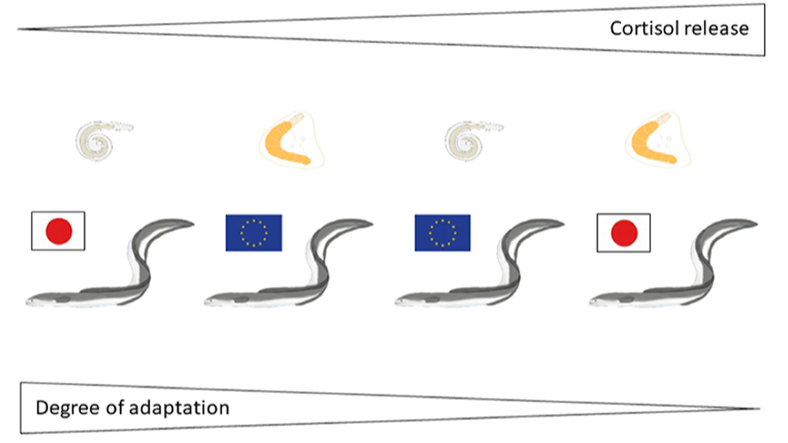

## 1 Introduction

Ecosystems are dynamic habitats with a variety of abiotic and biotic interactions. Among biotic components, invasive species often negatively affect native communities and change interactions within ecosystems (Simberloff and Rejmanek 2011). Although such invasive species are attaining increasing interest, their parasites are often neglected, even if co-introduced parasites affect new habitats occasionally to a greater extent than their hosts (Ballari et al., 2016; Keppel et al., 2014; Lymbery et al., 2014). However, invasive parasites do not only affect native hosts, but parasite communities can also be changed (Hatcher and Dunn, 2011; Hohenadler et al., 2019). Recently, two invasive parasites, *Anguillicola crassus* (Kuwahara, Niimi and Hagaki, 1974) and *Pomphorhynchus* sp. Monticelli, 1905, were shown to influence each other's establishment, supporting that the invasion meltdown hypothesis also applies to parasites (Hohenadler et al., 2018a). Specifically, the nematode *A. crassus* utilizes encapsulated cysts of the acanthocephalan *Pomphorhynchus* sp. as a hideout in a paratenic host (*Neogobius melanostomus* (Pallas, 1914)) to escape the fish’ immune response (Emde et al., 2014). Thereby *A. crassus* exploits an alternative way of infesting the European eel (*Anguilla anguilla* (Linnaeus, 1758)) as the definitive host (Hohenadler et al., 2018a).

The swim bladder nematode *A. crassus* was introduced to Europe in the early 1980s and ever since spread rapidly over the European eel population (Hartmann, 1993; Koops and Hartmann, 1989) and a few years later through American and African eel populations likewise (Barse et al., 2001; Sasal et al., 2008). This parasite is characterized by a long-lasting history of mutual adaptations with its native definitive host, the Japanese eel (*Anguilla japonica* Temmick and Schlegel, 1846), while all the other *Anguilla* spp., such as the European eel can be considered as non-adapted final hosts (Taraschewski, 2006). The long-lasting co-adaptations between Japanese eels and *A. crassus* lead to an effective immune response keeping the numbers of successfully establishing nematodes low. Moreover, almost no physiological consequences following an infestation in Japanese eels are known (Dangel et al., 2015; Keppel et al., 2014). Nevertheless, mutual adaptations allow the nematode to successfully infest its original host (Keppel et al., 2016). As these co-adaptations are missing in the European eel, the pathogenicity of *A. crassus* infestations in the new host is more severe and accompanied by a less effective immune defense of the host (Keppel et al., 2014; Knopf, 2006). One indication of the physiological imbalance within Japanese and European eels with *A. crassus* might be their stress response, which can be determined by analyzing their plasma cortisol concentrations after infestations with *A. crassus*. According to the current state of knowledge, infestations with *A. crassus* induce acute stress in European eels, throughout larval and young adult stages, in the form of increased plasma cortisol levels (Dangel et al., 2014; Sures et al., 2001). In contrast to laboratory infestations (Keppel et al., 2014; Sures et al., 2001), no cortisol increase for wild infested eels have been detected, which might be due to the fact that the duration of the infestation is unknown (Kelly et al., 2000). Consequences of increased cortisol levels are diverse: on short term it up-regulates the energy mobilization such as gluconeogenesis, on long term it down-regulates pathways, which cost a lot of energy, such as growth, reproduction and immune functions (Faught and Vijayan, 2016). These processes in turn might lead to higher susceptibility for secondary infections such as viruses, bacteria and other parasites (Sures, 2001; Sures et al., 2006).

Even though it is known that eels are unsuitable definitive hosts for species of the genus *Pomphorhynchus*, a high prevalence of immature acanthocephalans is usually found in eels (Thielen et al., 2007). *Pomphorhynchus* spp. prefer barbel (*Barbus barbus* (Linnaeus, 1758)) or chub (*Squalius cephalus* (Linnaeus, 1758)) as final hosts and do not mature in the European eel, which is therefore considered a dead-end host for these parasites (Sures et al., 2019). However, cystacanths of *Pomphorhynchus* sp. occur in the round goby (*Neogobius melanostomus*), which might be used as a paratenic host for the acanthocephalan. In addition, part of these *Pomphorhynchus* sp. cystacanths were found to harbor *A. crassus* larvae (Emde et al., 2014) allowing for a successful transmission of *A. crassus* to the European eel (Hohenadler et al., 2018a). The exact species of *Pomphorhynchus* sp. appearing in eels in the River Rhine remains unclear due to morphological similarities between *P. tereticollis* (Rudolphi, 1809), *P. laevis* (Zoega in Müller, 1776) and *P. bosniacus* Kiskároly and Čanković, 1967 which have frequently been misidentified (Emde et al., 2012; Hohenadler et al., 2018b; Reier et al., 2019). Nevertheless, species of the genus *Pomphorhynchus*, most likely *P. tereticollis* (Hohenadler et al., 2018b), are known for a long period to be present in eel populations (Sures et al., 1999; Thielen et al., 2007) but have most likely been replaced during the last 20–30 years (Sures et al., 2019). Hence, it seems reasonable that European eels might have developed adaptations to cope with infestations with this acanthocephalan genus to a greater extent than to infestations with *A. crassus*. Compared to European eels, no records of *Pomphorhynchus* sp. in Japanese eels are known (Amin et al., 2007; Katahira and Nagasawa, 2014; Leidy and Van Cleave, 1924; Van Cleave, 1925), which can therefore be considered as a presumably naïve system.

Since it appears that mutual adaptations of hosts and their parasites can be reflected in the stress response, we aimed to test the hypothesis that the stress response of eels against non-adapted parasites is higher than against adapted ones. We tested this hypothesis using the following differently adapted host-parasite systems: 1. Japanese eel and *A. crassus* (well-balanced mutual adaptations due to a long lasting history); 2. European eel and *Pomphorhynchus* sp. (presumably established mutual adaptations based on a rather long period of co-occurrence); 3. European eel and *A. crassus* (presumably weak mutual adaptations due to the recent invasion of *A. crassus* 40 years ago); and 4. Japanese eel and *Pomphorhynchus* sp. (no mutual adaptations due to missing co-occurrence).

## 2 Materials and Methods

### 2.1 Experimental design

**Fig. 1:**
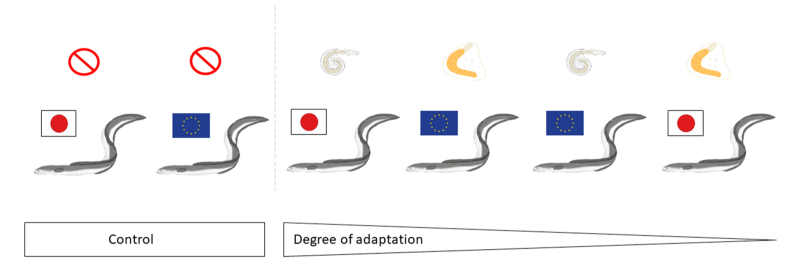
Overview of experimental eel groups.

To characterize the stress response of eels experimentally inoculated with *A. crassus* and *Pomphorhynchus* sp., fish were divided into six groups of ten individuals each (Fig. 1). One group of each eel species remained un-inoculated and served as a control, one group of each eel species was inoculated with 15 third stage larvae (L3) of *A. crassus* per eel, and the last group of each eel species was inoculated with 16–18 acanthocephalan cysts, partly containing *A. crassus* (see Hohenadler et al., 2018a). Both, the isolated L3 as well as the cysts were administered by a stomach tube to each eel. Therefore, eels were gently wrapped in a well-soaked cloth, and the respective number of larvae or cysts was administered by a stomach tube (1.5 mm diameter; B. Braun Melsungen AG, Melsungen, Germany) as described by Sures and Knopf (2004). Following inoculation, eels were maintained for 154 days in individual, aerated 30 l tanks using a flow through system and fed twice a week with eel pellets (DAN-EX 2848, BioMar A/S, Brande, Denmark) until parasitological examination. Plastic tubes served as hiding places in every tank.

### 2.2 Animal source

European and Japanese eels were obtained from eel farms known to be free of *A. crassus* (Albe Fischfarm, Haren/Rütenbrock, Germany and Omori-Tansui Co., Ltd., Miyazaki, Japan). To verify the absence of any endoparasites, initially ten eels of each species were randomly chosen, killed, dissected and screened with light microscopy for parasites. For experimental infestation of eels, eggs containing second stage larvae (L2) of *A. crassus* were collected from European eels caught by fishermen from the River Rhine. Development to the L3 stage was performed by offering copepods (*Macrocyclops albidus*) freshly hatched L2, cultured in the lab. Cysts of the acanthocephalan *Pomphorhynchus* sp. were collected from naturally infested invasive gobies (*Neogobius melanostomus*) provided by a professional fishermen as described in Hohenadler et al. (2018a). All experimental protocols were approved by the Ethics Council (Landesamt für Natur, Umwelt und Verbraucherschutz, Nordrhein-Westfalen, Germany, permit number: 84–02.04.2017.A245) and were carried out in accordance with the relevant guidelines and regulations.

### 2.3 Cortisol analyses

In order to measure plasma cortisol levels, blood samples of 150 μl were drawn from the caudal vein of each eel at 0, 28, 42, 70, 98 and 154 days post infestation (dpi). Eels were not sedated, considering that it took a maximum of 40 s between netting, drawing blood and transferring them back to the tank. If blood drawing was impossible within this time frame, eels were transferred back and no sample was taken at this occasion. Blood samples were allowed to clot for 2 h at room temperature and then centrifuged for 10 min at 5.000 g to separate serum from other blood parts. Only serum samples were frozen at −80 °C until further examination. Analyses of cortisol in eel sera were performed according to the manufacturer's instructions by an enzyme linked immunosorbent assay (Cortisol ELISA RE 52611, IBL International GmbH, Germany). Samples were transferred to microtiter plates coated with rabbit anti-cortisol antibodies. After the coloring reaction, optical density was measured at λ = 450 nm on a microplate reader (Tecan, infinite M200). Samples were measured in triplicates.

### 2.4 Statistical analysis

Graphs were plotted with Graphpad prism Version 8.4.1. Outliers have been removed by performing the ROUT test with Q = 1%. One individual of the control group of European eel had more than 50% of time points removed, so the complete individual was removed. In the groups of inoculation with L3-larvae only eels with *A. crassus* infestation were considered. In the groups of inoculation with *Pomphorhynchus* sp. cysts, all eels were considered regardless the underlying infestation with *A. crassus*.

## 3 Results

At the end of the experiment, direct administration of L3 of *A. crassus* resulted in a higher infestation rate in European eels than in Japanese eels (Table 1). Individuals of *Pomphorhynchus* sp. were not found in either eel species inoculated with the acanthocephalan cysts. However, individuals of *A. crassus* were identified in four eels of each species following administration of acanthocephalan cysts. Control groups of both eel species were free of *Pomphorhynchus* sp. and *A. crassus*. Details of the parasitological examination are shown in Table 1.

**Table 1:** Mean (±SD) intensities of *A. crassus* following experimental inoculation in European and Japanese eels.

Eel species	Type of inoculation	Stage of *A. crassus*			
		adult	preadult	L4	L3
European eel	isolated *A. crassus* (L3)	2.0 ± 0.8	5.0 ± 0.0	2.1 ± 1.0	1.6 ± 0.8
	encysted *Pomphorhynchus* sp.	1.5 ± 0.5	–	–	–
	uninfested control	–	–	–	–
Japanese eel	isolated *A. crassus* (L3)	2.0 ± 1.0	1.0 ± 1.0	2.5 ± 0.5	1.0^a^
	encysted *Pomphorhynchus* sp.	1.5 ± 0.5	–	–	–
	uninfested control	–	–	–	–

a : only one individual was found.

**Fig. 2:**
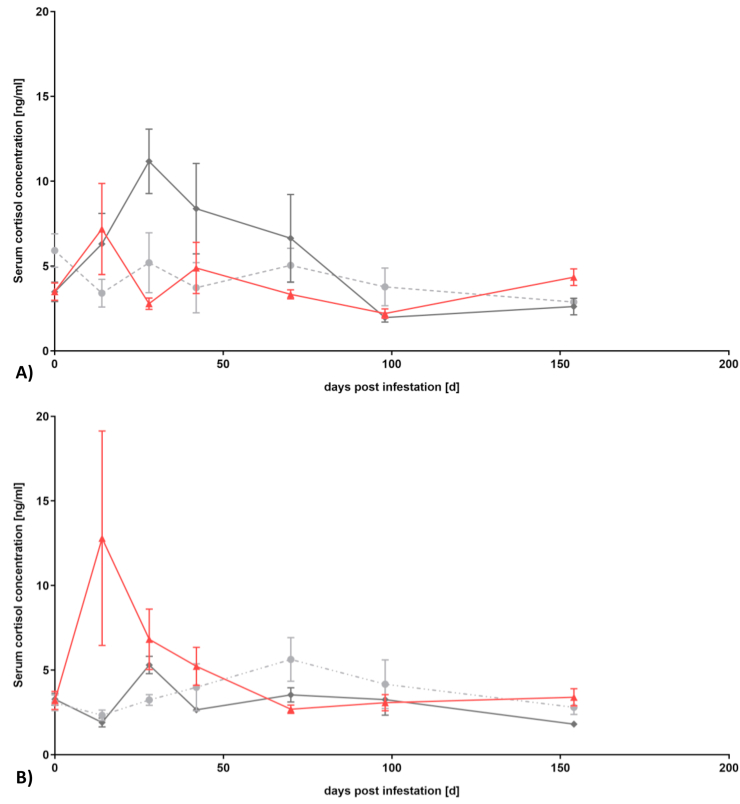
Course of mean cortisol concentrations (±SD) of A) European eels and B) Japanese eels. (red triangle = acanthocephalan cysts; dark grey square = L3 *A. crassus*; light grey circle = un-inoculated control).

To check the stress response, plasma cortisol levels of inoculated and untreated control eels were measured and the results are shown in Fig. 2.

The mean of serum cortisol concentration of European eels infested with L3 of *A. crassus* increased from 3.5 ± 1.6 ng/ml plasma cortisol at 0 dpi to 11.2 ± 5.4 ng/ml at 28 dpi. European eels inoculated with acanthocephalan cysts showed an increase of serum cortisol concentration from 3.5 ± 1.5 ng/ml at 0 dpi to 7.2 ± 7.1 ng/ml at 14 dpi. The group of Japanese eels infested with *A. crassus* shows an initial plasma cortisol level of 3.3 ± 0.9 ng/ml and an increase to 5.3 ± 1.2 ng/ml at 28 dpi. In the group inoculated with *Pomphorhynchus* sp. the plasma cortisol concentration was initially at 2.7 ± 0.9 ng/ml and increased to 9.5 ± 10.3 ng/ml at 14 dpi. The cortisol levels of un-inoculated control eels stayed constant during the whole experiment with a mean of 3.9 ± 2.5 ng/ml for European eels and 3.2 ± 1.9 ng/ml for Japanese eels. From 70 dpi onwards, cortisol levels of all groups of both eel species ranged in a similar range. Plasma cortisol concentrations for both eel species after inoculation with *Pomphorhynchus* sp. cysts were independent of a subsequent successful establishment of *A. crassus*.

## 4 Discussion

The present results clearly support our hypothesis that mutual adapted host-parasite systems are characterized by a lower stress level of the hosts compared to rather new host-parasite associations. The response of both eel species to infestations with *Pomphorhynchus* sp. cysts have not been investigated so far, whereas the cortisol values of both, the Japanese and the European eel, infested with *A. crassus* determined during the present study confirm previous results (Dangel et al., 2014; Sures et al., 2001). Taking a closer look on the results, all groups of the Japanese eel, including the un-inoculated control group, had a low starting value of plasma cortisol. The well adapted system of Japanese eels with *A. crassus,* showed only slightly increased plasma cortisol concentration after infestations with this parasite. In comparison to that, the group inoculated with *Pomphorhynchus* sp., which represents the group with presumably no mutual adaptations, showed the strongest cortisol increase by more than threefold compared to the initial plasma cortisol concentrations at 14 dpi. Comparing this to the results of European eels, an opposite pattern is evident. European eels started with a slightly higher mean cortisol concentration compared to Japanese eels. In the group of presumably weak adaptations with *A. crassus*, an almost three times higher cortisol release was detected at 28 dpi, whereas in the presumably established system with *Pomphorhynchus* sp. the cortisol concentration doubled at 14 dpi but then decreased again before remaining approximately at the cortisol level of the un-inoculated control eels. No effects of the *A. crassus* larvae encapsulated in the cysts of *Pomphorhynchus* sp. were detected on cortisol concentrations in any of the eel species. Accordingly, the idea “that the nematode larvae behave like immunological hitchhikers that follow a Trojan horse strategy in order to avoid the paratenic host's immune response” (Hohenadler et al., 2018a) obviously also applies to other physiological processes, i.e. in avoiding a stress response by the host.

The high standard deviations obtained at some days can most likely be attributed to individual differences, which were also described earlier (Dangel et al., 2014; Silva et al., 2018; Sures et al., 2006). However, despite a comparably high SD, clear patterns emerge, suggesting a stress response for less mutually adapted host-parasite systems.

Consequences of increased cortisol levels as shown here are diverse. On short term cortisol up-regulates the energy mobilization such as gluconeogenesis, on long term it down-regulates pathways which cost a lot of energy, such as growth, reproduction and immune functions (Faught and Vijayan, 2016). Consequences of *A. crassus* infestations on eels in general are well known; various data reveal an influence on the swim bladder function, an increase of stress parameters and therefore a decrease of the eel's immune system, which makes the eels more susceptible to secondary infections with viruses, bacteria or other parasites (Barry et al., 2014; Kennedy, 2007; Kirk, 2003; Schneebauer et al., 2017; Würtz et al., 1996; Würtz and Taraschewski, 2000). Therefore, some of the known consequences of *A. crassus* infestations in European eels might be related to increased cortisol expression (Sures et al., 2006, 2001).

Whereas *A. crassus* is a highly specific eel parasite, the eel is considered a dead-end host for *Pomphorhynchus* sp. - even though the acanthocephalan is able to establish and start growing in this fish species (Hohenadler et al., 2018b). To the best of our knowledge, consequences of *Pomphorhynchus* sp. infestations to either of the eel species were never investigated. Furthermore, the appearance of *Pomphorhynchus* sp. was only described in European eels, but never for Japanese eels (Katahira and Nagasawa, 2014; Nagasawa and Katahira, 2017; Thielen et al., 2004). Van Cleave described a single appearance of *Acanthocephalus gotoi* sp. Van Cleave, 1925 in Japanese eels from fish markets, nearly 100 years ago (Van Cleave, 1925). More recent studies described acanthocephalan infestations of giant mottled eels (*Anguilla marmorata* Quoy and Gaimard, 1824) from Japan (Katahira and Nagasawa, 2014) and infestations of the Japanese eel with a single individual of *Echinorhynchus cotti* Yamaguti, 1939 and *Pseudorhadinorhynchus samegaiensis* Nakajima and Egusa, 1975 (Amin et al., 2007). In European eels, some acanthocephalan species are known to be host-specific such as *Acanthocephalus anguillae* (Müller, 1780) and *Paratenuisentis ambiguus* (Van Cleave, 1921), as they also mature in European eels (Kennedy, 2006; Taraschewski et al., 1987). Species of the genus *Pomphorhynchus* can survive in European eels, where they are commonly found, but as they never reach maturity, eels are not a suitable definitive host for this parasite (Bates and Kennedy, 1991; Thielen et al., 2004).

The cortisol release of Japanese eels to infestations with the acanthocephalan cysts and the European eel to infestations with *A. crassus* indicates that both eel species showed similar stress responses to unknown parasites. In contrast, slightly or fully adapted parasites do not influence the cortisol response of their hosts. The pattern of a lower cortisol release for better adapted host-parasite-systems has also been observed for other species. For example, the ectoparasite *Caligus rogercresseyi* Boxshall and Bravo, 2000 affects the Chilean salmon industry as it infests primarily Atlantic salmon (*Salmo salar* Linnaeus, 1758), but not Coho salmon (*Oncorhynchus kisutch* (Walbaum, 1792)), which appears to be immune to infestation by this crustacean (Valenzuela-Muñoz et al., 2016). Comparative studies of these host-parasite systems also demonstrated that the better adapted *O. kisutch* has a considerably lower cortisol release than the less well adapted *S. salar* following infestation with *C. rogercresseyi* (Vargas-Chacoff et al., 2019).

The results of the present study as well as of previous investigations on salmon parasites suggests that the stress response of the host can be used to indicate differently adapted host-parasite systems. The well-balanced mutual adaptations between the Japanese eel and *A. crassus* do not lead to a measureable stress response of the host following inoculation with the parasite. In contrast, the cortisol response of the Japanese eel to inoculation with *Pomphorhynchus* sp. was the highest cortisol release during the experiment, which might indicate a complete lack of adaptation due to missing co-occurrence under natural conditions. The cortisol response of the European eel - as well as their degree of adaptation with the parasites chosen - ranges between that of the Japanese eel. Since nearly 40 years, *A. crassus* and the European eel co-occur in the River Rhine. This period might not be enough to lead to a mutual adaptation as can be seen by a rather strong cortisol release. Even if European eels are not suitable final host for *Pomphorhynchus* sp. they do have a long history of co-occurrence with some species such as *P. tereticollis* (Hohenadler et al., 2018b; Sures et al., 2019) what might provide them with some adaptations, which is also reflected in a relatively weak cortisol release compared to *A. crassus* infestations.

## 5 Conclusions

Invasive species must adapt to their newly conquered ecosystem - the same is valid for parasites and their new hosts. Mutual adaptations determine the success of the invasion process. Following inoculation of eel hosts with parasite larvae, the cortisol release relates negatively to the degree of adaptation. Specifically, highly adapted systems such as the Japanese eel with *A. crassus* showed no cortisol response in contrast to systems with no adaptation at all, such as the Japanese eel following inoculation with cystacanths of *Pomphorhynchus* sp., which showed by far the strongest cortisol response of all investigated host-parasite systems investigated within this study.

## Declaration of competing interest

The authors declare that they have no known competing financial interests or personal relationships that could have appeared to influence the work reported in this paper.
